# 2D cine vs. 3D self-navigated free-breathing high-resolution whole heart cardiovascular magnetic resonance for aortic root measurements in congenital heart disease

**DOI:** 10.1186/s12968-021-00744-1

**Published:** 2021-05-27

**Authors:** Clément Nussbaumer, Judith Bouchardy, Coralie Blanche, Davide Piccini, Anna-Giulia Pavon, Pierre Monney, Matthias Stuber, Jürg Schwitter, Tobias Rutz

**Affiliations:** 1 Service of Cardiology, Centre de Resonance Magnétique Cardiaque, Lausanne University Hospital and University of Lausanne, Lausanne, Switzerland; 2 Service of Cardiology, Adult Congenital Heart Disease Unit, Lausanne University Hospital and University of Lausanne, Lausanne, Switzerland; 3 Department of Radiology, University Hospital and University of Lausanne, Lausanne, Switzerland; 4 Advanced Clinical Imaging Technology, Siemens Healthcare AG, Lausanne, Switzerland

**Keywords:** Aortic root dilatation, 3D self-navigation whole heart, Congenital heart disease, Bicuspid aortic valve

## Abstract

**Background:**

Cardiovascular magnetic resonance (CMR) is considered the method of choice for evaluation of aortic root dilatation in congenital heart disease. Usually, a cross-sectional 2D cine stack is acquired perpendicular to the vessel’s axis. However, this method requires a considerable patient collaboration and precise planning of image planes. The present study compares a recently introduced 3D self-navigated free-breathing high-resolution whole heart CMR sequence (3D self nav) allowing a multiplanar retrospective reconstruction of the aortic root as an alternative to the 2D cine technique for determination of aortic root diameters.

**Methods:**

A total of 6 cusp-commissure (CuCo) and cusp-cusp (CuCu) enddiastolic diameters were measured by two observers on 2D cine and 3D self nav cross-sectional planes of the aortic root acquired on a 1.5 T CMR scanner. Asymmetry of the aortic root was evaluated by the ratio of the minimal to the maximum 3D self nav CuCu diameter. CuCu diameters were compared to standard transthoracic echocardiographic (TTE) aortic root diameters.

**Results:**

Sixty-five exams in 58 patients (32 ± 15 years) were included. Typically, 2D cine and 3D self nav spatial resolution was 1.1–1.5^2^ × 4.5-7 mm and 0.9–1.15^3^ mm, respectively. 3D self nav yielded larger maximum diameters than 2D cine: CuCo 37.2 ± 6.4 vs. 36.2 ± 7.0 mm (p = 0.006), CuCu 39.7 ± 6.3 vs. 38.5 ± 6.5 mm (p < 0.001). CuCu diameters were significantly larger (2.3–3.9 mm, p < 0.001) than CuCo and TTE diameters on both 2D cine and 3D self nav. Intra- and interobserver variabilities were excellent for both techniques with bias of -0.5 to 1.0 mm. Intra-observer variability of the more experienced observer was better for 3D self nav (F-test p < 0.05). Aortic root asymmetry was more pronounced in patients with bicuspid aortic valve (BAV: 0.73 (interquartile (IQ) 0.69; 0.78) vs. 0.93 (IQ 0.9; 0.96), p < 0.001), which was associated to a larger difference of maximum CuCu to TTE diameters: 5.5 ± 3.3 vs. 3.3 ± 3.8 mm, p = 0.033.

**Conclusion:**

Both, the 3D self nav and 2D cine CMR techniques allow reliable determination of aortic root diameters. However, we propose to privilege the 3D self nav technique and measurement of CuCu diameters to avoid underestimation of the maximum diameter, particularly in patients with asymmetric aortic roots and/or BAV.

**Supplementary Information:**

The online version contains supplementary material available at 10.1186/s12968-021-00744-1.

## Background

**Fig. 1:**
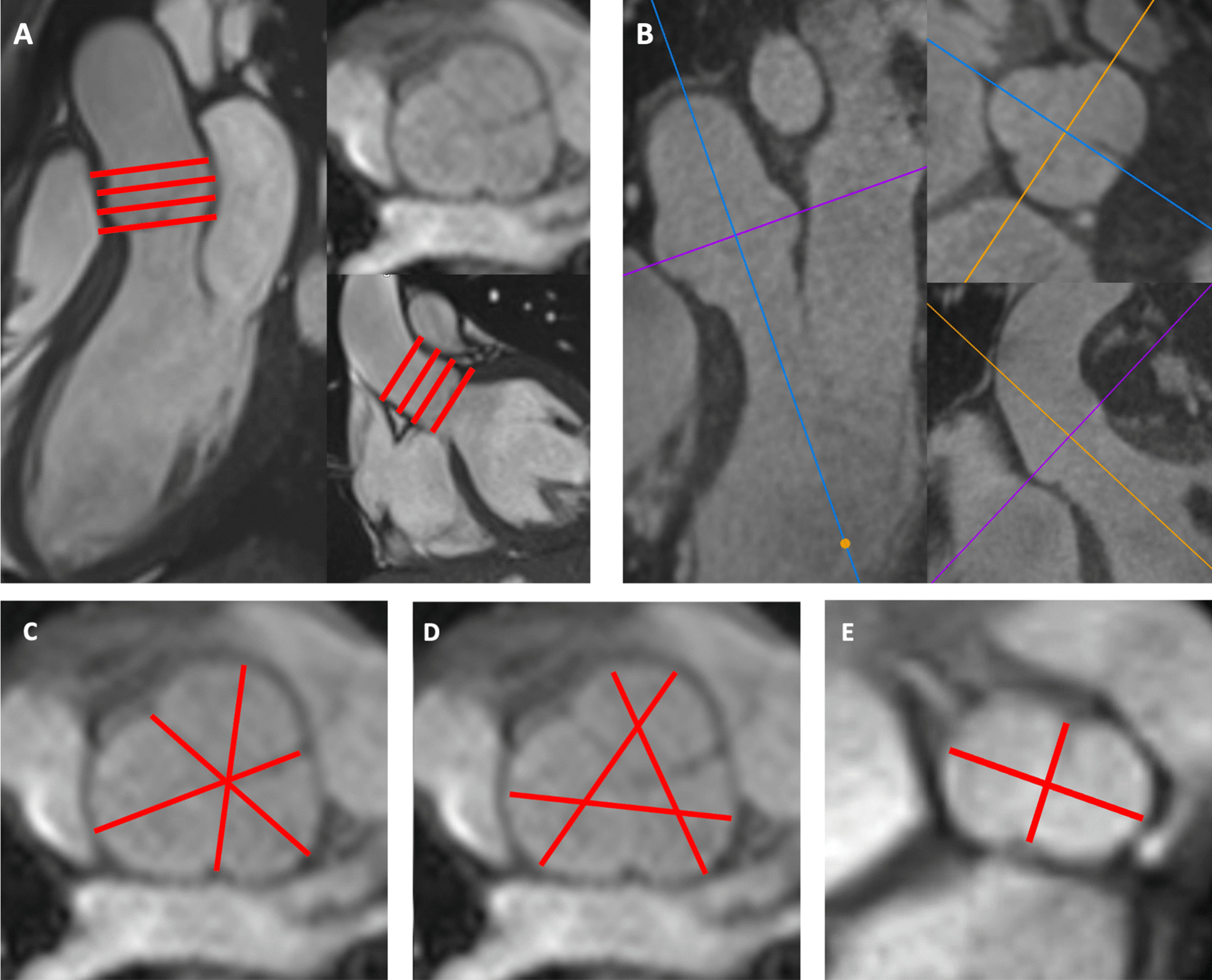
The doubly-oblique cross-sectional stack of 2D cines was planned on two orthogonal cines of the left ventricular outflow tract (LVOT)  (**a**). For 3D self nav, a multiplanar reconstruction of the aortic root plane was performed (**b**). Cusp to the opposite commissure (CuCo) (**c**) and cusp to cusp (CuCu, **d**) inner to inner edge diameters were determined. For bicuspid aortic valve (BAV) patients in whom identification of three cusps was not possible, the maximum diameter and a second, orthogonal diameter were obtained (**e**)

Dilatation of the ascending aorta is frequently encountered in congenital heart disease (CHD) like bicuspid aortic valve (BAV) and hereditary aortopathies such as Marfan, Loeys-Dietz or Turner syndrome [1–4]. Regular imaging of the aorta is essential in these patients to time prophylactic surgical intervention for prevention of dramatic consequences such as aortic dissection [5–8]. To correctly evaluate the vessel diameters perpendicular to the vessel axis, three-dimensional (3D) imaging techniques like cardiovascular magnetic resonance (CMR) or cardiac computed tomography (CCT) are used. Bright and dark blood CMR techniques have been introduced for assessment of the aorta. Black blood T1-and T2-weighted sequences allow assessment of the aorta and its wall without the administration of a contrast agent [9, 10]. Typical bright blood sequences are the contrast-enhanced angiography, providing a 3D data set of the aorta, and the fast gradient echo (GRE) or the balanced steady-state free precession (bSSFP) sequences, the two latter sequences not requiring contrast administration. As the aortic root presents an important pulsatility and translational motion of up to 2 cm during the cardiac cycle, electrocardiogram (ECG)-gated imaging techniques are required to avoid blurring of the vessel wall [11, 12]. There is, however, no uniform method to measure aortic root diameters and practice variation exists with regard to determination of leading to leading (L-L), inner to inner (I-I) or outer to outer (O–O) edge diameters as well as measurements of cusp to commissure (CuCo) or cusp to cusp (CuCu) diameters [11, 13, 14]. When using CMR, a stack of 2D cines is prescribed in doubly-oblique cross-sectional orientation on two orthogonal long axis cines of the left ventricular (LV) outflow tract (LVOT) and perpendicular to the axis of the aortic root (Fig. 1a) [14]. This technique requires, however, a careful planning of the 2D cine stack to limit variability of measurements. In addition, repetitive breath-holds during the acquisition of the 2D cines are necessary, which can be difficult for populations with suboptimal compliance such as patients with trisomy 21, intellectual deficit or dyspnea. An alternative is the use of a 3D self-navigated free-breathing high-resolution whole heart CMR sequence (3D self nav) with either end-systolic or diastolic gating [15]. This technique, which has been introduced by Piccini et al. in 2012, uses a radial readout extracting the respiratory motion data directly at the level of the heart and from the k-space data [16–18]. The use of a 3D radial readout allows achieving a high isovolumetric spatial resolution as well as acquiring the self-navigating readout at each heart beat while maintaining uniformity in the sampling. The trajectory has a kooshball like arrangement of the readouts, in which every single k-space line crosses the center of k-space. The geometrical distribution of the readouts follows a spiral phyllotaxis patterns as described previously [18]. The advantage of the latter method is a free-breathing image acquisition without the need for meticulous scan plane adaptations and respiratory navigator placement, thus leading to a relatively simple exam with 100% scan efficiency and a predictable image acquisition duration, all of which rendering the exam very comfortable for the patient [17, 19]. The acquired high-resolution 3D whole heart volume allows a flexible retrospective multiplanar reconstruction (MPR) of the image plane perpendicular to the vessel’s axis for determination of the aortic root diameters.

The aim of the present study was therefore to compare the precision and the reliability of both the 2D cine and 3D self nav methods in determining aortic root diameters in CHD patients with different aortic pathologies.

## Methods

This is a retrospective study on CMR exams performed for aortic root evaluation at a single institution. The study was approved by the local ethics committee with waiver of informed consent.

### Population

Patients undergoing CMR for evaluation of the aortic root between 2014 and 2019 by both types of CMR sequences, 2D cine and 3D self nav, were included. Both sequences were part of the routine imaging protocol in these patients. Patients with data of only one of the two above-mentioned CMR sequences were excluded.

### CMR Imaging

Patients were scanned with two 1.5 T clinical CMR scanners (MAGNETOM Aera and Sola, Siemens Healthineers, Erlangen, Germany) with a 30-channel phased-array coil. The imaging protocol was selected according to the specific malformation. [14, 20] Contrast medium was used in all patients (Gadobutrol, Gadovist®, Bayer Healthcare, Berlin, Germany, dose: 0.2 mmol/kg). No heart rate lowering medication was used.

### 2D cine CMR Imaging

To acquire the 2D cine stack in doubly-oblique, cross-sectional orientation to the aortic root axis, first a standard 3-chamber long axis cine of the was obtained [20]. Second, an LVOT cine was prescribed orthogonal to the 3-chamber view. Third, a stack of eight to ten 2D cines without gap of the aortic root was planned perpendicular to the aortic root axis on both, the 3 chamber and LVOT cines (Fig. 1a). 2D cines were acquired using either a fast gradient echo (GRE) or a bSSFP sequence. Use of GRE was chosen by the physician in charge of the CMR exam in case of relevant image artifacts on bSSFP. Typical imaging parameters for GRE and bSSFP sequences were matrix 256/146, slice thickness 4.5–7 mm, in-plane resolution 1.1–1.5 mm, temporal resolution < 50 ms, duration of breath-holding about 8 to 12 s. Specific parameters for GRE were TR/TE 51/3.5 ms, flip angle 15°; for bSSFP TR/TE 45/1.3 ms, flip angle 59°.

### 3D self-navigated free-breathing high-resolution whole heart CMR

The prototype 3D self nav sequence was acquired as previously described for CHD patients [15]. The 3D self nav CMR sequence was started after injection of the contrast agent for the 3D contrast-enhanced angiography. The imaging parameters were as follows: TR/TE 3.1/1.56 ms, field-of-view 200–220^3^ mm, matrix 192^3^, receiver bandwidth 900 Hz/pixel, isotropic spatial resolution (both acquired and reconstructed) 0.9–1.15 mm, about 12′000 radial readouts, acquisition duration 8–10 min. The trigger delay was visually identified at the most quiescent mid- to end-diastolic period on a cine 4 chamber view.

### CMR image analyses

Two observers, one with < 1-year experience in CMR (NC, observer 1), and one experienced reader (TR, observer 2, EuroCMR level III certified in general and congenital CMR), performed the measurements of aortic root diameters. They were blinded to diagnosis and previous medical surgery of patients. Prior to the aortic diameter measurements, observer 1 trained for about 20 h to analyze the CMR images under the supervision of observer 2.

### CMR image quality

Image quality was assessed for each CMR acquisition technique according to a five-point scale as previously described [15]: Grade 5 corresponds to an excellent and grade 4 to a good diagnostic quality with mild blurring, whereas grade 3 indicates diagnostic quality, despite moderate blurring of cardiac and vascular structures. Grade 2 indicates marked blurring of the structures, preventing a complete anatomical diagnosis. In grade 1, a dataset was considered non-diagnostic.

### CMR aortic root diameter measurement

Aortic root measurements were performed on Syngo.Via ™ (Siemens Healthineers). For measurement on 2D cines, each observer chose the adequate slice of the 2D cine stack representing the maximal aortic diameter at end-diastole (Fig. 1a). For 3D self nav measurements, each observer performed a MPR of the image plane representing the largest aortic root diameters (Fig. 1b). For both, 2D cine and 3D self nav, a total of six I-I diameters were measured: three CuCo and three CuCu diameters (Fig. 1c, d). Diameters were classified as minimal, mid and maximum diameter according to their measured length. For 18 (28%) patients with apparent “real” BAV, identification of three cusps was not possible on 2D cine and 3D self nav. For these patients, the maximal diameter and a second, orthogonal diameter were obtained as previously described (Fig. 1e). [21] Of note, for 3 patients, identification of three cusps was only possible on one imaging technique (observer 1: two patients on 3D self nav, one patient on 2D cine; observer 2: 1 patient on 3D self nav). Observer 1 performed both, CuCo and CuCu measurements twice for all patients. Observer 2 performed the same measurements twice for 20 randomly chosen patients allowing to assess the inter-observer variability and the intra-observer variabilities for both observers.

An asymmetry index of the aortic root was calculated by obtaining the ratio of the minimal to the maximum 3D self nav CuCu diameter.

### Transthoracic echocardiographic (TTE) aortic root diameter measurement

Results of 2D aortic root measurements were obtained from TTE exams performed within 6 months of the CMR. In our institution, a standard 2D parasternal long-axis view is obtained with a zoom on the aortic root. The end-diastolic frame is chosen, and the diameter of the aortic root measured using the L-L technique as proposed by the guidelines of the American Society of Echocardiography (ASE) and European Association of Cardiovascular Imaging (EACVI). [11].

### Statistical analysis

Normality was tested with the Shapiro–Wilk test. Parameters are indicated as mean ± standard deviation (SD) or as number and percentage, where appropriate. Wilcoxon signed-rank test was used to assess differences in the scoring of image quality. Bland–Altman analyses were performed to compare 2D cine and 3D self nav diameters. [22] Bland–Altman and intraclass correlation coefficient (ICC) analyses were performed to evaluate intra- and inter-observer variabilities of diameter measurements of 2D cine and 3D self nav. Paired t-tests or Wilcoxon signed-rank tests, where appropriate, were performed for comparison of continuous parameters and F-test for comparison of variances. Pearson’s correlation was used to determine the correlation between parameters.

Influence of baseline parameters (image quality, use of GRE, BAV, prior aortic valve surgery, presence of a genetic syndrome, AI) on comparisons of 2D cine and 3D self nav diameters, intra- and inter-observer variabilities were determined by t-tests or Pearson’s correlation. Statistical analysis was performed using SPSS (version 26.0, Statistical Package for the Social Sciences, International Business Machines, Inc., Armonk, New York, USA), Excel (version 16.35, Microsoft, Redmond, Washington, USA) and Graphpad Prism (version 5, Graphpad Software, San Diego, California, USA).

## Results

### Study population

Sixty-five CMR exams from 58 patients were included (reasons for two CMR exams in 7 patients are indicated in Additional file 1). Baseline characteristics of the population are shown in Table 1. Patient population consisted of four groups: non-operated aortic pathologies (BAV 28%, coarctation of the aorta 5%, subvalvular aortic membrane 3%), prior aortic valve or root surgery (Ross procedure 22%, Tirone David Procedure 8%, commissurotomy 8%, Hemashield Graft 3%, Bentall procedure 2%), syndromic patients (Marfan syndrome 14%, Turner syndrome 3%, Ehlers-Danlos’ syndrome 2%) and patients undergoing CMR for work-up of the ascending aorta (5%). GRE was used in 19 (29%) of exams.

**Table 1:** Patients’ characteristics

	Total cohort	
	N = 65	
*Patient characteristics*		
Age	Years	32.3 ± 14.6
Male gender	N (%)	42 (64.6%)
Height	cm	174.7 ± 11.5
Weight	kg	69.5 ± 16.8
BSA	m^2^	1.83 ± 0.24
Arterial hypertension	N (%)	13 (20.0%)
*Patient diagnosis*		
Prior aortic root surgery	N (%)	27 (41.5%)
Time since surgery	Years	12.3 ± 7.9
Non-operated aortic pathologies	N (%)	23 (35.4%)
Genetic syndrome	N (%)	12 (18.5%)
Ascending aorta assessment	N (%)	3 (4.6%)
*Aortic valve characteristics*		
Bicuspid aortic valve	N (%)	24 (36.9%)
CMR aortic regurgitant fraction	%	9.4 ± 12.4
Aortic regurgitation by TTE	N (%)	45 (69.2%)
Grade 1		26 (40.0%)
Grade 2		14 (21.5%)
Grade 3		5 (7.7%)
Aortic stenosis	N (%)	12 (18.5%)
Maximal aortic gradient	mmHg	12.3 ± 10.7
Mean aortic gradient	mmHg	6.2 ± 5.5
Maximal aortic velocity	cm/s	158.0 ± 66.3
*Aortic diameters*		
Aortic root diameter on TTE	mm	36.0 ± 7.9
CMR^a^		
Ascending aorta level of right pulmonary artery	mm/m^2^	17.5 ± 4.5
Aortic arch	mm/m^2^	12.5 ± 2.4
Descending aorta level of diaphragm	mm/m^2^	± 1.9

*BSA* body surface area, *TTE* transthoracic echocardiography

^a^Measured on either a standard contrast-enhanced 3D angiography or 3D self nav

### CMR image quality

**Fig. 2:**
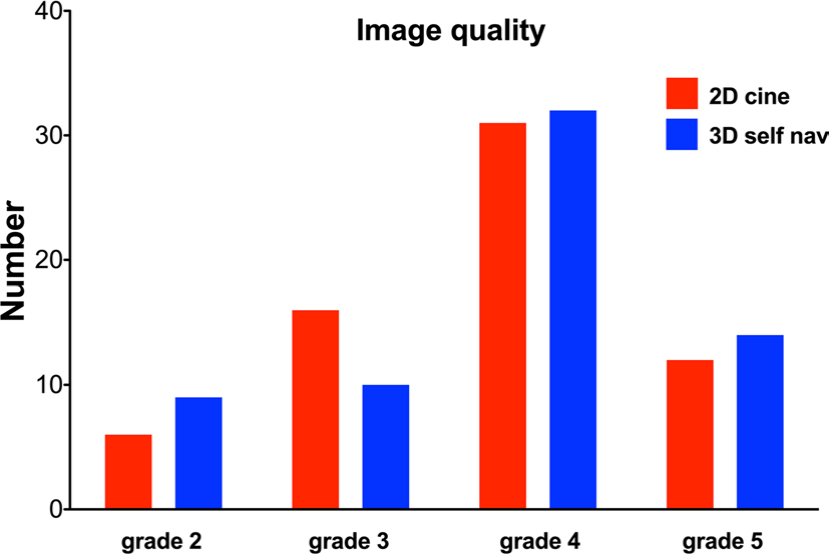
Comparison of image quality of 2D cine (red bars) and 3D self nav (blue bars). There were no differences of image quality neither between techniques nor between observers (p > 0.05)

The image quality did not differ between 2D cine and 3D self nav (p = 0.857, Fig. 2) nor between both observers: *p* = 0.705 for 2D cine and *p* = 0.221 in 3D self nav.

### Aortic root measurement

**Fig. 3:**
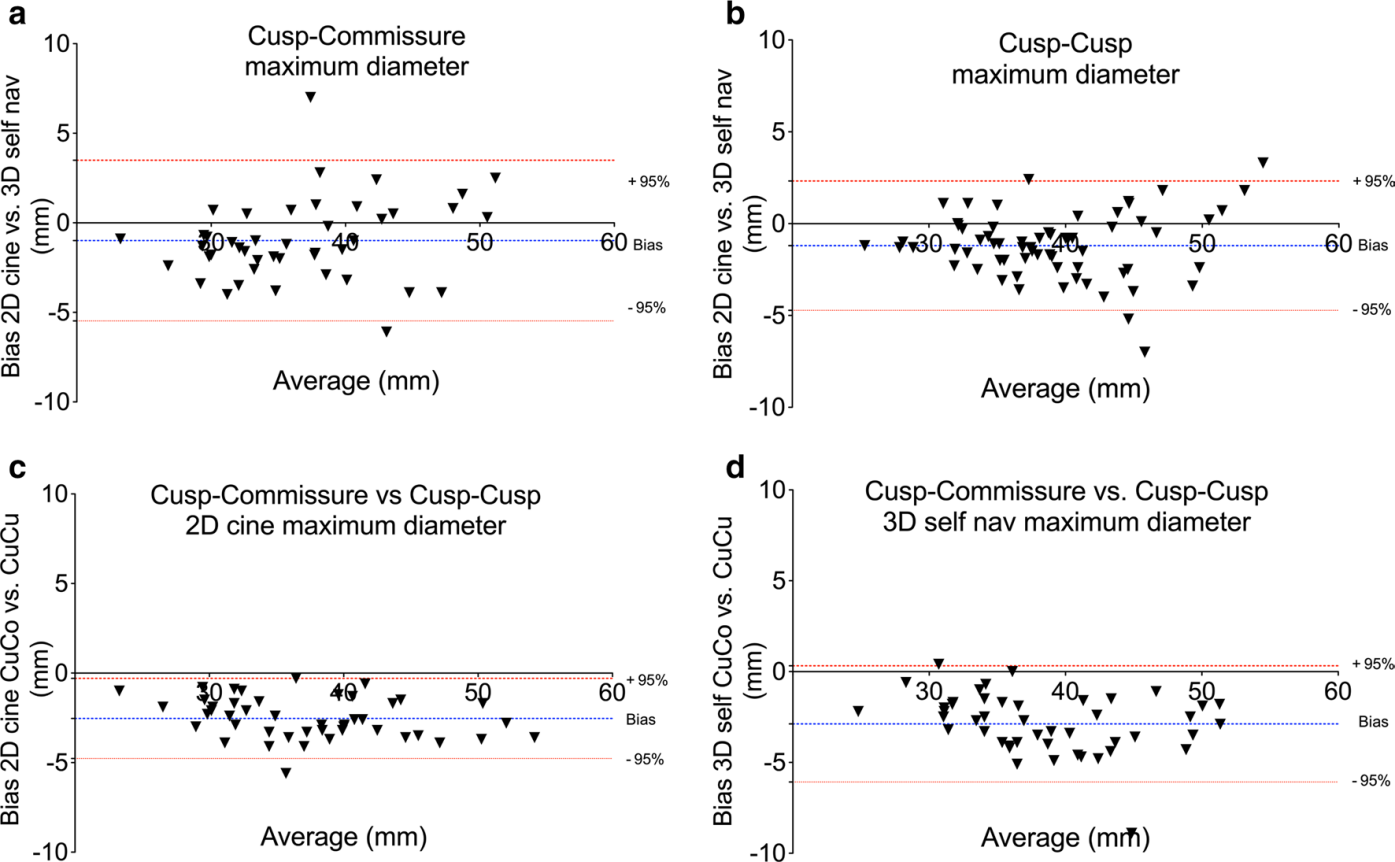
Upper panels: Bland–Altman plots for the comparison of the maximum cusp to commissure (CuCo) (**a**) and cusp to cusp (CuCu) (**b**) diameters between 2D cine vs. 3D self nav. Lower panels: Comparison maximum CuCo to CuCu diameters measured on 2D cine (**c**) and 3D self nav (**d**). The blue line represents the mean bias and the doted red lines indicate the 95% confidence interval. *CuCo* cusp to commissure, *CuCu* cusp to cusp

Although Pearson’s correlation analyses showed a strong correlation of diameters obtained by both, 2D cine and 3D self nav (r = 0.94–0.97), aortic root diameters were systematically larger on 3D self nav compared to 2D cine (range = 0.8–1.3 mm for observer 1, p < 0.01, Table 2 and Table S1). Figure 3a, b show the Bland–Altman plots for the comparison of the maximum CuCo and CuCu diameters measured on 2D cine vs. 3D self nav.

**Table 2:** Comparison of maximum 2D cine CMR vs 3D self nav CMR aortic root diameters

2D cine vs. 3D self nav	Observer 1		Observer 2	
	CuCo max	CuCu max	CuCo max	CuCu max
Mean diameter 2D cine (mm)	36.2	38.5	35.8	38.2
Mean diameter 3D self nav (mm)	37.2	39.7	37.3	39.5
Mean difference (mm)	− 1.0	− 1.2	− 1.6	− 1.3
95% Limits of agreement (mm)	− 5.5 to 3.5	− 4.7 to 2.3	− 4.7 to 1.4	− 5.0 to 2.4
Standard deviation (mm)	2.3	1.8	1.5	1.9
Variance (mm^2^)	5.2	3.2	2.0	3.6
Pearson’s correlation (r)	0.945	0.951	0.985	0.964
P value (t-test)	0.006	< 0.001	0.001	0.009

*3D self nav* 3D self-navigated high-resolution free-breathing whole heart, *CuCo* cusp to commissure, *CuCu* cusp to cusp, *max* maximum

CuCu diameters were systematically 2.3 to 2.9 mm larger than the CuCo diameters on both 2D cine and 3D self nav (*p* < 0.001). Detailed results are presented in Table 3 and Fig. 3c, d. Results on comparison of minimal and mid diameters between 2D cine and 3D self nav as well as CuCu and CuCo are provided in Additional file 1: Tables S1, S2).

**Table 3:** Comparison of maximum Cusp to Commissure vs. Cusp to Cusp diameters

	2D cine	3D self nav
Mean diameter CuCo (mm ± SD)	36.2 ± 6.9	37.0 ± 6.3
Mean diameter CuCu (mm ± SD)	38.7 ± 7.2	39.9 ± 6.8
Bias (mm)	− 2.5	− 2.9
Standard deviation (mm)	1.1	1.6
95% Limits of agreement (mm)	− 4.8; − 0.3	− 6.1; 0.3
Variance (mm^2^)	1.3	2.6
Pearson’s correlation (r)	0.988	0.972
P value (t-test)	< 0.001	< 0.001

*CuCo* cusp to commissure, *CuCu* cusp to cusp

Intra-observer variabilities were excellent for both techniques with a mean bias of ≤ 0.6 mm for both observers and both, the 2D cine and 3D self nav techniques (Table 4 and Table S3, Additional file 1). ICC values were excellent for both observers. For observer 1, variances were smaller for determination of maximum CuCo 2D cine vs. 3D self nav diameters. For observer 2, variances were smaller on 3D self nav for both, maximum CuCo and CuCu diameters (F test, *p* < 0.05). Bland–Altman plots of intra-observer variabilities of both observers for maximum CuCo and CuCu diameters are provided in Additional file 1.

**Table 4:** Intra-observer variability

	Observer 1		Observer 2	
	CuCo max	CuCu max	CuCo max	CuCu max
*2D cine*				
Mean difference (mm)	− 0.4	− 0.3	− 0.1	− 0.3
95% Limits of agreement (mm)	− 3.0 to 2.1	− 2.7 to 2.1	− 3.9 to 3.6	− 3.5 to 2.9
Standard deviation (mm)	1.3	1.2	1.9	1.6
Variance (mm^2^)	1.7	1.5	3.6	2.6
ICC	0.989	0.991	0.975	0.985
*3D self nav*				
Mean difference (mm)	− 0.6	− 0.6	− 0.1	− 0.4
95% Limits of agreement (mm)	− 5.3 to 4.0	− 3.4 to 2.2	− 1.9 to 2.0	− 2.3 to 1.5
Standard deviation (mm)	2.4	1.4	1.0	1.0
Variance (mm^2^)	5.6	2.0	1.0	1.0
ICC	0.965	0.971	0.994	0.994
P value	0.884	0.268	0.712	0.993
p-value (F test)	0.047	0.336	0.020	0.034

*ICC* interclass correlation coefficient, others see Table 3

The inter-observer variability was excellent with mean differences ranging from 0.1 mm to 0.8 mm for 2D cine and − 0.3 mm to 1.0 mm for 3D self nav, respectively (for maximum diameters see Table 5 and Additional file 1: Figure S3, for minimal and mid diameters see Additional file 1: Table S4). ICC values were excellent, and bias and variances did not differ significantly (p > 0.05).

**Table 5:** Interobserver variability

	CuCo max	CuCu max
*2D cine*		
Mean difference (mm)	0.5	0.6
95% Limits of agreement (mm)	− 2.9 to 3.9	− 2.7 to 3.8
Standard deviation (mm)	1.7	1.6
Variance (mm2)	3.0	2.7
ICC	0.966	0.983
*3D self nav*		
Mean difference (mm)	− 0.3	1.0
95% Limits of agreement (mm)	− 6.4 to 5.8	− 2.6 to 4.5
Standard deviation (mm)	3.1	1.8
Variance (mm2)	9.7	3.2
ICC	0.950	0.978
P value (t-test)	0.484	0.495
p-value (F test)	0.139	0.910

Abbreviations: see Tables 3 and 4

TTE aortic root diameters were available for all but two patients. While maximum 2D cine and maximum 3D self nav CuCo diameters did not differ significantly to maximum TTE aortic root diameters, maximum CuCu diameters were significantly larger on both, 2D cine and 3D self nav measurements with larger biases for diameters measured on 3D self nav (Table 6). The differences between TTE and maximum CuCu diameters were larger in BAV patients (5.5 ± 3.3 vs. 3.3 ± 3.8 mm, p = 0.033). Of note, the decision on identification of three cusps did not match in 3 cases for observer 1 and in one case for observer 2. As patients without three identified cusps were allocated to the CuCu measurements and for two patients no TTE diameters were available, resulting TTE diameters in Table 6 differ slightly between analyses when using paired t-tests.

**Table 6:** Comparison of aortic root diameters between echocardiography and cardiac magnetic resonance

TTE vs. 2D cine	CuCo max	CuCu max	TTE vs. 3D self nav	CuCo max	CuCu max
Observer 1			Observer 1		
Mean diameter TTE (mm)	37.2	36.0	Mean diameter TTE (mm)	37.0	36.0
Mean diameter 2D cine (mm)	36.6	38.4	Mean diameter 3D self nav (mm)	37.4	39.9
Mean difference (mm)	− 0.6	2.3	Mean difference (mm)	0.4	3.9
Limits of agreement (mm)	− 7.4 to 6.2	− 4.4 to 9.1	Limits of agreement (mm)	− 8.0 to 8.7	− 3.5 to 11.3
Standard deviation (mm)	3.5	3.4	Standard deviation (mm)	4.2	3.8
Variance (mm^2^)	12.1	11.8	Variance (mm^2^)	18.0	14.3
Pearson’s correlation (r)	0.972	0.903	Pearson’s correlation (r)	0.882	0.881
P value (t-test)	0.247	< 0.001	P value (t-test)	0.564	< 0.001
Observer 2			Observer 2		
Mean diameter TTE (mm)	36.9	36.6	Mean diameter TTE (mm)	36.6	36.6
Mean diameter 2D cine (mm)	36.1	38.4	Mean diameter 3D self nav (mm)	37.8	39.9
Mean difference (mm)	− 0.8	1.8	Mean difference (mm)	1.1	3.4
Limits of agreement (mm)	− 9.7 to 8.2	− 6.0 to 9.6	Limits of agreement (mm)	− 5.9 to 8.2	− 3.1 to 9.9
Standard deviation (mm)	4.6	4.0	Standard deviation (mm)	3.6	3.3
Variance (mm^2^)	20.8	15.9	Variance (mm^2^)	12.8	10.9
Pearson’s correlation (r)	0.881	0.869	Pearson’s correlation (r)	0.932	0.914
P value (t-test)	0.532	0.065	P value (t-test)	0.221	< 0.001

Of note, the decision on identification of three cusps did not match in 3 cases for observer 1 and in one case for observer 2. As patients without three identified cusps were allocated to the CuCu measurements and for two patients no TTE diameters were available, resulting TTE diameters in Table 6 differ slightly between analyses when using paired t-tests

*TTE* transthoracic echocardiography, other see Tables 3 and 4

Significant aortic root asymmetry was found in 15 (23%) of included patients when using an asymmetry index cut-off of <0.75. AI was significantly smaller in BAV than in non-BAV patients: 0.73 (interquartile (IQ) 0.69; 0.78) vs. 0.93 (IQ 0.9; 0.96), p < 0.001). A more severe asymmetry was associated with a larger discrepancy of TTE aortic root diameters to maximum CuCu 3D self nav diameter: AI < 0.75 (N = 15) vs. ≥ 0.75 (N = 48) = 5.6 ± 3.4 vs. 3.4 ± 3.8 mm, p = 0.047. Of note, all but one patient with an asymmetry index < 0.75 were BAV patients.

### Further supplementary material

We evaluated parameters influencing the precision of 2D cine and 3D self nav measurements. There was no significant impact of image quality or the use of GRE on comparison of 2D cine vs. 3D self nav measurements nor on intra- or inter-observer variabilities (*p* > 0.05). Aortic root surgery, asymmetry index, maximum aortic root diameter, presence of BAV and genetic syndrome influenced the bias and reliability of some diameter determinations, for details please see tables S5-7 in Additional file 1. Of note that aortic root surgery did not influence the image quality.

## Discussion

This study systematically compares two CMR sequences for aortic root evaluation in patients with CHD. We provide four important findings to physicians performing CMR in this patient population:

1. 2D cine and 3D self nav are both reliable methods for aortic root measurements with only small differences in precision.
2. 3D self nav provides significantly larger diameters than 2D cine.
3. CuCu diameters are significantly larger than CuCo diameters on both techniques.
4. TTE diameters are systemically smaller than the maximum CuCu diameters. The difference is highly influenced by the aortic root asymmetry and the presence of BAV.

### Precision of 2D cine and 3D self nav

Although aortic root dilatation has an important impact on mortality and morbidity in CHD patients, guidelines on image acquisition, choice and measurement of aortic root diameters are relatively vague [6, 8, 23]. Due to the important pulsatility and throughplane motion of the aortic root, ECG-gated imaging techniques are required [12]. Studies on precision of different CMR aortic root evaluation techniques are, however, surprisingly scarce [24–27]. Burman et al. determined normal values for aortic root diameters in healthy individuals using sagittal, coronal and perpendicular cross-sectional 2D cines of the aortic root suggesting the use of cross-sectional 2D cine and measurement of CuCo diameters [25]. Unfortunately, this very elegant study did not provide information on the precision of the applied imaging and measurement methods. Several studies compared non-triggered angiography with 2D cine or 3D bSSFP whole heart CMR, the latter usually ECG triggered and using a respiratory navigator [24, 26, 28]. For the 2D cine technique, a relatively large range of biases of 0.2 to 4 mm was reported for inter- and intra-observer variabilities [21, 26, 27, 29]. Further studies compared 3D bSSFP whole heart sequences using respiratory navigators to either 3D ECG gated angiography or TTE [24, 30, 31]. Veldhoen et al. did not observe differences for the precision of aortic root measurement by CMR 3D angiography and ECG gated 2D bSSFP, which is surprising but probably explained by the fact that only Marfan patients after aortic root replacement were included. One can speculate that the pulsatility and throughplane motion of the aortic root is diminished after surgery explaining why a non-triggered sequence like an 3D angiography performed relatively well [24]. Potthast showed that 3D bSSFP whole heart performed best for vessel diameter determination when compared to 3D contrast-enhanced CMR angiography, 2D T2 black blood, and 2D cine bSSFP [10].

All the above-cited studies used 3D bSSFP sequences with pencil beam respiratory navigators for respiratory motion correction. As an alternative, 3D self-navigation techniques have been developed operating without a reference position and extracting the respiratory motion at the heart and from the k-space data [17, 19, 32]. The 3D self nav sequence used in this study offers a high spatial resolution with an isovolumetric voxel size of about 1.1 mm^3^ in contrast to most studies using 2D or 3D bSSFP sequences and its acquisition duration is predictable [10, 24, 29, 33]. The potential of the 3D self nav has been shown for imaging of coronary arteries, evaluation of anatomy in CHD and detection of myocardial scar [15, 17, 34]. In the present study, the image quality was comparable between both sequences (Fig. 2) and, with respect to the 3D self nav sequence, similar to what our group has previously published [15]. Intra- and inter-observer variabilities were excellent as expressed by a small bias of ≤ 1 mm and small limits of agreements comparable to previously published reports on reproducibility of 2D cine and 3D whole heart techniques [3, 15, 24, 26]. We observed a better reproducibility for the 3D self nav method for the maximum diameter determination for the experienced observer. This finding is probably explained by the fact that performing MPRs on 3D self nav in complex anatomy is challenging for less experienced observers in contrast to “simply” choosing the 2D cine slice representing the largest diameter. Of note that all CMR exams were performed by physicians experienced in CMR (EuroCMR level III certified), assuring the correct orientation of 2D cines, which may have contributed to the high agreement observed between 2D cine and 3D self nav.

Diameters were systematically about 1 mm smaller on 2D cines compared to 3D self nav. Two factors probably explain this observation. First, the lower spatial resolution (both, inplane resolution and slice thickness) of 2D cines compared to the 3D self nav, although the 2D cine spatial resolution was similar to previous studies [25]. The resulting blurring of the aortic wall due to the partial volume effect results in an underestimation of the maximum diameters. Second, the timing of the 3D self nav to the most-quiescent diastolic period i.e. the mid to the end-diastolic phase as well as the higher temporal resolution of the 2D cine, might also contribute to the slightly larger diameters obtained by the 3D self nav sequence.

The precision of 2D cine and 3D self nav was influenced neither by the image quality nor using GRE for the 2D cine sequence. Aortic root surgery and AI were associated with a better agreement of 2D cine and 3D self nav diameters for observer 1 but not for observer 2. This might be explained by the fact that the aortic root presents less pulsatility and throughplane motion after surgery reducing the variability between both methods.

Identification of the aortic root diameter appears to be more challenging in asymmetric roots, explaining the influence of asymmetry index, the maximum aortic root diameter, BAV and genetic syndrome on some, however, only few measurements.

### Which aortic root diameter should be measured by which method?

Most long-standing recommendations on aortic disease are historically based on TTE L-L measurements. Several recent international and national guidelines tried to uniform the recommendations on aortic root evaluation [5, 11, 13, 14, 35, 36]. The 2015 ASE/EACVI guidelines for chamber quantification suggest for CMR and CT both CuCo and CuCu measurements [11]. However, this same document as well as the 2014 European Society of Cardiology guidelines on aortic disease elucidate that it was not possible to obtain uniformity between different imaging modalities and currently, recommendations vary suggesting CuCu, CuCo as well as I-I, L-L and O–O diameters and different image planes and sequences [5, 11]. A further problem is that in studies identifying cut-offs for surgical intervention in e.g. Marfan, BAV or Tuner patients, either multiple imaging modalities and measurement techniques were used or information on diameter assessment are lacking [6, 23, 37–39].

Burman et al. found similar to our study significantly larger CuCu than CuCo diameters [25]. They suggested to privilege CuCo diameters as they correlated best to age and body surface area and were found to correspond closely to reference TTE root measurements recorded in the Framingham Heart Study cohort. However, based on our observations, this method is associated with a considerable risk of underestimation of the largest aortic root diameter. We show that CMR CuCu diameters are systematically larger than TTE and up to 3 mm larger than CuCo diameters. Both observations are in line with previous studies on healthy individuals, BAV and CHD patients [21, 25, 26]. Particularly in BAV patients, TTE is at risk to underestimate the maximum diameter due to an increased aortic root asymmetry. The asymmetry was significantly more important in BAV patients than in non-BAV patients and associated to a larger discrepancy of CuCu diameters to TTE diameters (5.5 vs. 3.3 mm), similar to previous findings [21, 26]. In contrast to TTE, CT and CMR show a very good agreement when determining I-I aortic root diameters as performed in our study [21, 29, 38].

There is therefore a clear need for harmonization and a prospective validation of imaging and measurement protocols as well as thresholds for surgical intervention.

Based on our findings, we have adopted the strategy to measure aortic root diameters on the 3D self nav whole heart images which are routinely acquired during our CHD CMR scan protocol. This strategy reduces the scan time by about 10 min as no acquisition of a second LVOT and the cross-sectional cines of the aortic root is required [14, 20]. In addition, as the 3D self nav sequence allows the evaluation of the whole cardiac and thoracic anatomy and vessels it can replace the anatomic localizers and the 3D angiography [15].

### Limitations

As it is a retrospective study, the choice of slice thickness of the 2D cines was to the discretion of the physician in charge, the reason why it was not uniform and varied between 4.5 to 7 mm, but without influence on precision (p > 0.05). The heterogeneity of the patient population could be criticized, however, showing also the robustness of both techniques in different pathologies and conditions and, e.g. demonstrating that aortic root evaluation in BAV patients is prone to underestimation of the correct maximum diameter when using CuCo diameters. Decision on the contrast media administration was to the discretion of the physician in charge of the exam. In our institution, a contrast-enhanced 3D CMR angiography is performed for all patient who undergo, in particular if for the first time, a CMR exam for assessment of the great vessels. This explains why contrast media was administered for all patients, which, however, would not be required for the sole assessment of aortic root diameters. The 3D self nav technique may also perform well without contrast as previously shown [15].

### Conclusions

Both, the 2D cine and the 3D self nav technique are reliable methods for determination of aortic root diameters in CHD patients. Considering the higher spatial resolution and a more convenient acquisition for the patients, the 3D self nav technique could be privileged for the follow-up of patients with aortic root enlargement. In addition, CuCu diameters should be determined as, particularly in asymmetric aortic roots like in BAV patients, CuCo diameters present a considerable risk to underestimate the maximum diameter.

## Supplementary Information

**Additional file 1. **Additional figures and tables.

## Data Availability

The datasets used and/or analyzed during the current study are available from the corresponding author on reasonable request.

## Abbreviations

3D self nav: 3D self-navigated free-breathing high-resolution whole heart sequence
BAV: Bicuspid aortic valve
bSSFP: Balanced steady-state free precession
CCT: Cardiac computed tomography
CHD: Congenital heart disease
CMR: Cardiovascular magnetic resonance
CuCo: Cusp to commissure
CuCu: Cusp to cusp
ECG: Electrocardiogram
GRE: Gradient echo
HR: Heart rate
L-L: Leading to leading
LV: Left ventricle/left ventricular
LVOT: Left ventricular outflow tract
MPR: Multiplanar reconstruction
O-O: Outer to outer
TTE: Transthoracic echocardiography
SD: Standard deviation
