# Supplementary material for: 2D cine vs. 3D self-navigated free-breathing high-resolution whole heart cardiovascular magnetic resonance for aortic root measurements in congenital heart disease
Source: J Cardiovasc Magn Reson. 2021 May 27;23:65. doi: 10.1186/s12968-021-00744-1 (PMC8157643; doi:10.1186/s12968-021-00744-1)
Supplement: Supplementary file 1 — Additional file 1. Additional figures and tables. [file 12968_2021_744_MOESM1_ESM.docx]

**Reasons for two CMR exams in 7 patients:**

One Marfan patient for regular follow-up, one Marfan patient with close follow-up because of aortic root diameters close to indications for aortic root replacement, one Ross patient with severe aortic root dilatation and bad transthoracic image quality, one bicuspid aortic valve (BAV) patient with a regular 4 year follow-up CMR, one Ross patient before and after right to pulmonary artery homograft replacement for endocarditis, one BAV patient with aortic root diameters close to indications for aortic root replacement, one Marfan patient with progressive aortic root dilatation requiring close follow-up.

**Figures S 1-3**

**Figure S1**


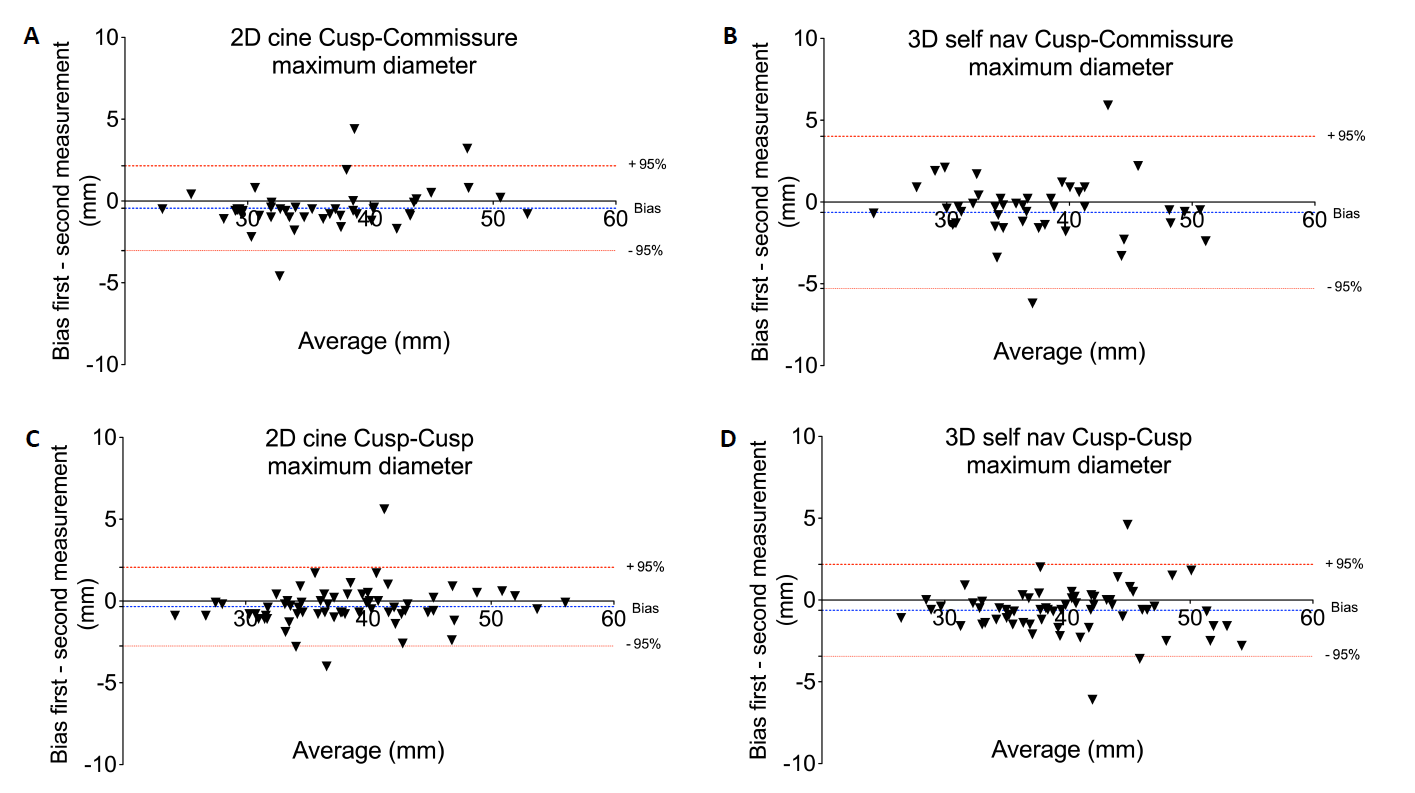


Bland-Altmann plots of the intra-observer variabilities for the 2D cine and 3D self nav cusp to commissure (CuCo) (A and B) and cusp to cusp (CuCu) (C and D) diameter measurements for observer 1. The blue line represents the mean bias and the doted red lines indicate the 95% confidence interval. CuCo = cusp to commissure, CuCu = cusp to cusp

**Figure S2**

**
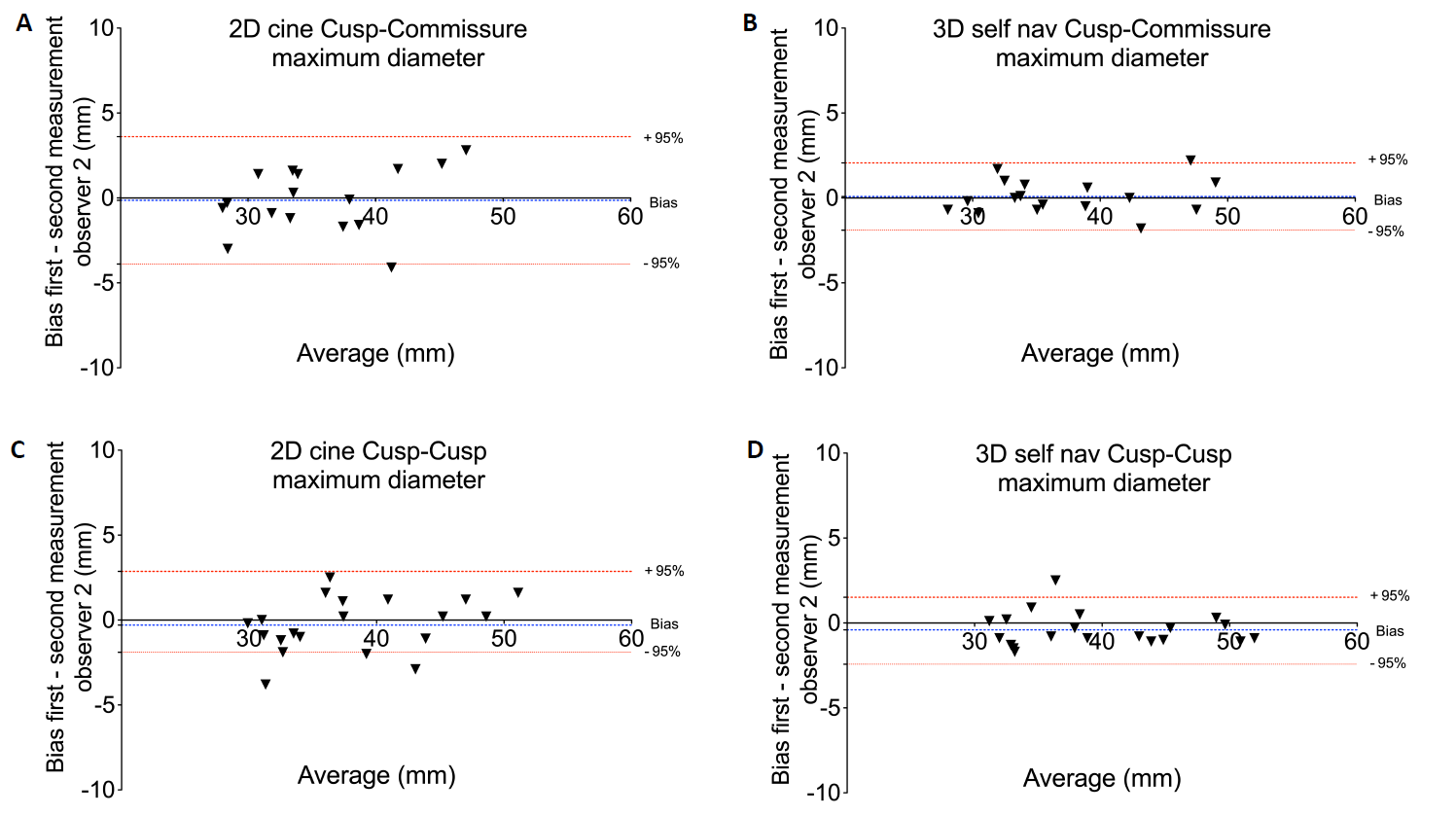
**

Bland-Altmann plots of the intra-observer variabilities for the 2D cine and 3D self nav CuCo (A and B) and CuCu (C and D) diameter measurements for observer 2. The blue line represents the mean bias and the doted red lines indicate the 95% confidence interval. CuCo = cusp to commissure, CuCu = cusp to cusp

**Figure S3**

**
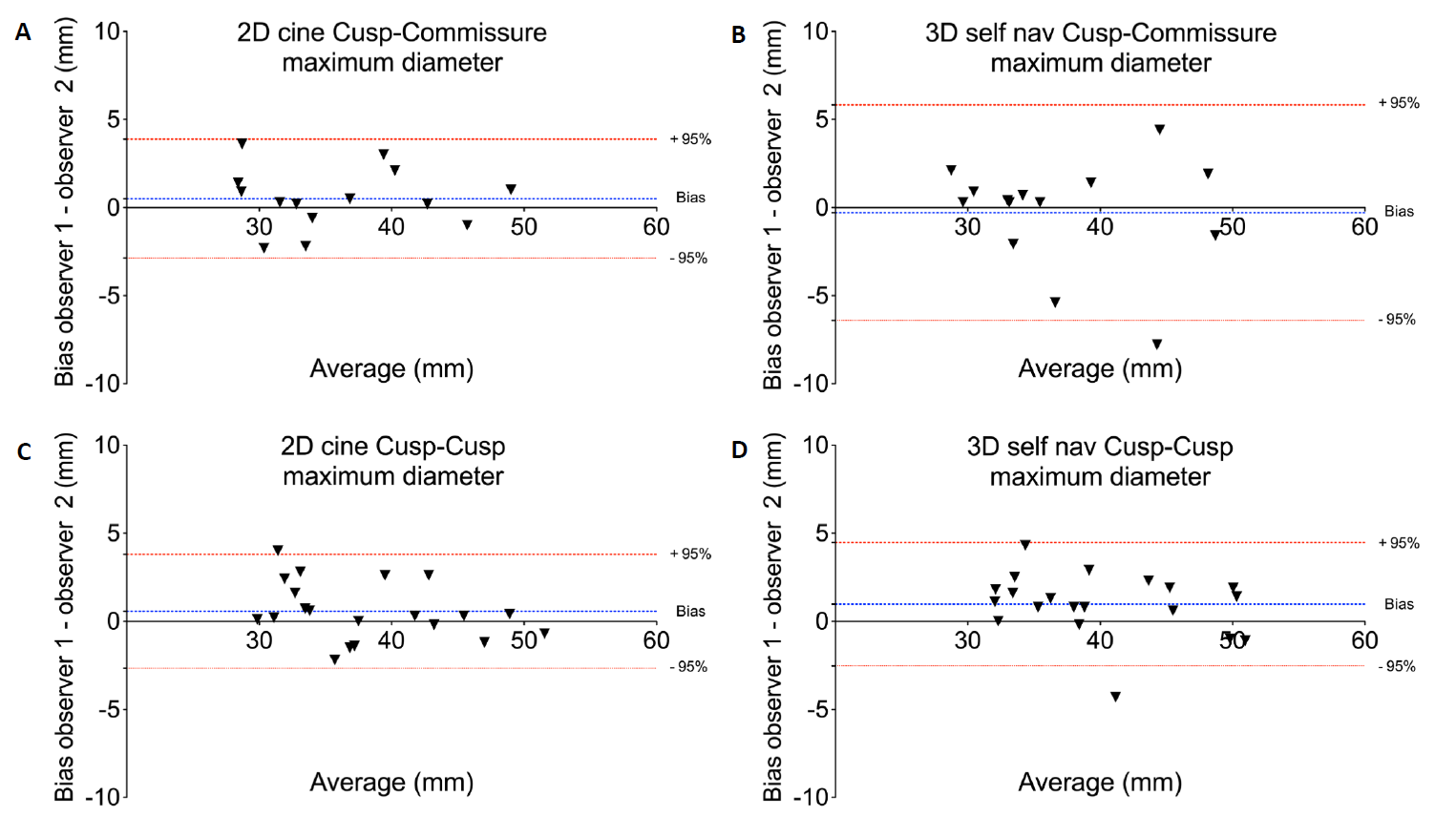
**

Bland-Altmann plots of the inter-observer variabilities for the 2D cine and 3D self nav CuCo (A and B) and CuCu (C and D) diameter measurements. The blue line represents the mean bias and the doted red lines indicate the 95% confidence interval. CuCo = cusp to commissure, CuCu = cusp to cusp

**Tables S1-7**

| **Table S1** Comparison of 2D cine vs 3D self nav aortic root diameters | | | | | | |
| --- | --- | --- | --- | --- | --- | --- |
| 2D cine vs. 3D self nav | CuCo min | CuCo mid | CuCo max | CuCu min | CuCu mid | CuCu max |
| **Observer 1** |  |  |  |  |  |  |
| Mean diameter 2D cine (mm) | 33.5 | 34.8 | 36.2 | 33.4 | 37.6 | 38.5 |
| Mean diameter 3D self nav (mm) | 34.5 | 35.9 | 37.2 | 34.3 | 38.5 | 39.7 |
| Mean difference (mm) | -1.0 | -1.1 | -1.0 | -0.8 | -1.3 | -1.2 |
| 95% Limits of agreement (mm) | -5.1 to 3.2 | -5.3 to 3.1 | -5.5 to 3.5 | -5.5 to 3.8 | -4.7 to 2.0 | -4.7 to 2.3 |
| Standard deviation (mm) | 3.2 | 2.1 | 2.3 | 2.4 | 1.7 | 1.8 |
| Variance (mm^2^) | 4.5 | 4.5 | 5.2 | 5.6 | 2.9 | 3.2 |
| Pearson’s correlation (r) | 0.952 | 0.954 | 0.945 | 0.944 | 0.972 | 0.951 |
| P value (t-test) | 0.003 | 0.001 | 0.006 | 0.005 | <0.001 | <0.001 |
| **Observer 2** |  |  |  |  |  |  |
| Mean diameter 2D cine (mm) | 32.8 | 34.3 | 35.8 | 33.9 | 36.9 | 38.2 |
| Mean diameter 3D self nav (mm) | 33.5 | 35.6 | 37.3 | 34.9 | 38.3 | 39.5 |
| Mean difference (mm) | -0.6 | -1.3 | -1.6 | -1.0 | -1.4 | -1.3 |
| 95% Limits of agreement (mm) | -3.8 to 2.5 | -3.0 to 0.5 | -4.7 to 1.4 | -4.6 to 2.6 | -5.9 to 3.1 | -5.0 to 2.4 |
| Standard deviation (mm) | 1.6 | 0.9 | 1.5 | 2.6 | 2.3 | 1.9 |
| Variance (mm^2^) | 2.9 | 0.8 | 2.0 | 3.4 | 5.3 | 3.6 |
| Pearson’s correlation (r) | 0.967 | 0.992 | 0.985 | 0.956 | 0.900 | 0.964 |
| P value (t-test) | 0.125 | <0.001 | 0.001 | 0.025 | 0.013 | 0.009 |
| Abbreviations: 3D self nav = 3D self-navigated high-resolution free-breathing whole heart, CuCo = cusp to commissure, CuCu = commissure to commissure, max = maximum, min = minimal | | | | | | |

| **Table S2** Comparison of Cusp to Commissure vs. Cusp to Cusp diameters | | | | | | |
| --- | --- | --- | --- | --- | --- | --- |
|  | **2D cine** | | | **3D self nav** | | |
| CuCo vs. CuCu | Minimal diameter | Mid diameter | Maximum diameter | Minimal diameter | Mid diameter | Maximum diameter |
|  |  |  |  |  |  |  |
| Mean diameter CuCo (mm ± SD) | 33.5 ± 6.7 | 34.8 ± 6.8 | 36.2 ± 6.9 | 34.3 ± 6.0 | 35.7 ± 6.0 | 37.0 ± 6.3 |
| Mean diameter CuCu (mm ± SD) | 35.7 ± 6.9 | 37.2 ± 7.1 | 38.7 ± 7.2 | 36.9 ± 6.5 | 38.4 ± 6.6 | 39.9 ± 6.8 |
| P value (t-test) | < 0.001 | < 0.001 | < 0.001 | < 0.001 | < 0.001 | < 0.001 |
| Bias (mm) | -2.3 | -2.4 | -2.5 | -2.6 | -2.7 | -2.9 |
| Standard deviation (mm) | 1.1 | 1.3 | 1.1 | 1.5 | 1.5 | 1.6 |
| 95% Limits of agreement (mm) | -4.5; -0.1 | -5.0; 0.2 | -4.8; -0.3 | -5.6; 0.4 | -5.6; 0.3 | -6.1; 0.3 |
| Variance (mm^2^) | 1.3 | 1.8 | 1.3 | 2.3 | 2.3 | 2.6 |
| Pearson’s correlation (r) | 0.987 | 0.983 | 0.988 | 0.973 | 0.975 | 0.972 |
|  | | | | | | |

Abbreviations: see table S2

| **Table S3** Intra-observer variability | | | | | | |
| --- | --- | --- | --- | --- | --- | --- |
|  | CuCo min | CuCo mid | CuCo max | CuCu min | CuCu mid | CuCu max |
| **Observer 1** |  |  |  |  |  |  |
| **2D cine** |  |  |  |  |  |  |
| Mean difference (mm) | -0.3 | -0.2 | -0.4 | -0.5 | -0.5 | -0.3 |
| 95% Limits of agreement (mm) | -2.7 to 2.2 | -2.8 to 2.4 | -3.0 to 2.1 | -4.0 to 3.0 | -2.7 to 1.7 | -2.7 to 2.1 |
| Standard deviation (mm) | 1.3 | 1.3 | 1.3 | 1.8 | 1.1 | 1.2 |
| Variance (mm^2^) | 1.6 | 1.7 | 1.7 | 3.2 | 1.3 | 1.5 |
| ICC | 0.991 | 0.990 | 0.989 | 0.982 | 0.992 | 0.991 |
| **3D self nav** |  |  |  |  |  |  |
| Mean difference (mm) | -0.3 | -0.3 | -0.6 | -0.3 | -0.6 | -0.6 |
| 95% Limits of agreement (mm) | -3.4 to 2.9 | -3.6 to 3.0 | -5.3 to 4.0 | -2.9 to 2.4 | -3.4 to 2.2 | -3.4 to 2.2 |
| Standard deviation (mm) | 1.6 | 1.7 | 2.4 | 1.4 | 1.4 | 1.4 |
| Variance (mm^2^) | 2.5 | 2.9 | 5.6 | 1.8 | 2.0 | 2.0 |
| ICC | 0.983 | 0.980 | 0.965 | 0.991 | 0.987 | 0.971 |
| P value | 0.518 | 0.770 | 0.884 | 0.802 | 0.762 | 0.268 |
| p-value (F test) | 0.563 | 0.210 | 0.047 | 0.684 | 0.402 | 0.336 |
| **Observer 2** |  |  |  |  |  |  |
| **2D cine** |  |  |  |  |  |  |
| Mean difference (mm) | -0.4 | -0.3 | -0.1 | -0.3 | -0.2 | -0.3 |
| 95% Limits of agreement (mm) | -3.5 to 2.6 | -2.9 to 2.3 | -3.9 to 3.6 | -4.3 to 3.6 | -3.6 to 3.2 | -3.5 to 2.9 |
| Standard deviation (mm) | 1.6 | 1.3 | 1.9 | 2.0 | 1.7 | 1.6 |
| Variance (mm^2^) | 2.4 | 1.8 | 3.6 | 4.1 | 3.0 | 2.6 |
| ICC | 0.983 | 0.988 | 0.975 | 0.974 | 0.985 | 0.985 |
| **3D self nav** |  |  |  |  |  |  |
| Mean difference (mm) | -0.4 | -0.1 | -0.1 | -0.2 | -0.3 | -0.4 |
| 95% Limits of agreement (mm) | -2.7 to 2.0 | -2.0 to 1.9 | -1.9 to 2.0 | -2.3 to 2.0 | -2.6 to 1.9 | -2.3 to 1.5 |
| Standard deviation (mm) | 1.2 | 1.0 | 1.0 | 1.1 | 1.2 | 1.0 |
| Variance (mm^2^) | 1.4 | 1.0 | 1.0 | 1.2 | 1.3 | 1.0 |
| ICC | 0.990 | 0.994 | 0.994 | 0.993 | 0.994 | 0.994 |
| P value | 0.923 | 0.467 | 0.712 | 0.728 | 0.711 | 0.993 |
| p-value (F test) | 0.355 | 0.259 | 0.020 | 0.100 | 0.182 | 0.034 |

Abbreviation: ICC = interclass correlation coefficient, others see table 3

| **Table S4** Inter-observer variability | | | | | | |
| --- | --- | --- | --- | --- | --- | --- |
|  | CuCo min | CuCo mid | CuCo max | CuCu min | CuCu mid | CuCu max |
| **2D cine** |  |  |  |  |  |  |
| Mean difference (mm) | 0.3 | 0.4 | 0.5 | 0.1 | 0.8 | 0.6 |
| 95% Limits of agreement (mm) | -2.7 to 3.4 | -3.7 to 4.4 | -2.9 to 3.9 | -4.8 to 4.9 | -3.0 to 4.5 | -2.7 to 3.8 |
| Standard deviation (mm) | 1.6 | 2.0 | 1.7 | 2.5 | 1.9 | 1.6 |
| Variance (mm2) | 2.4 | 4.2 | 3.0 | 6.2 | 3.7 | 2.7 |
| ICC | 0.987 | 0.976 | 0.966 | 0.958 | 0.981 | 0.983 |
| **3D self nav** |  |  |  |  |  |  |
| Mean difference (mm) | 0.5 | 0.2 | -0.3 | -0.3 | 0.9 | 1.0 |
| 95% Limits of agreement (mm) | -3.4 to 4.4 | -4.5 to 4.8 | -6.4 to 5.8 | -6.4 to 5.7 | -2.5 to 4.4 | -2.6 to 4.5 |
| Standard deviation (mm) | 1.9 | 2.4 | 3.1 | 3.1 | 1.8 | 1.8 |
| Variance (mm2) | 3.9 | 5.6 | 9.7 | 9.5 | 3.1 | 3.2 |
| ICC | 0.973 | 0.964 | 0.950 | 0.991 | 0.943 | 0.978 |
|  |  |  |  |  |  |  |
| P value (t-test) | 0.835 | 0.810 | 0.484 | 0.576 | 0.881 | 0.495 |
| p-value (F test) | 0.792 | 0.302 | 0.139 | 0.661 | 0.758 | 0.910 |
|  | | | | | | |

Abbreviations: see table 3 and 4

| **Table S5** Univariate analyses of parameters influencing bias 2D cine vs. 3D self nav |
| --- |

|  | **No (mm ± SD)** | **Yes (mm ± SD)** | ***p*** |
| --- | --- | --- | --- |
| **Observer 1** |  |  |  |
| **BAV** |  |  |  |
| CuCu minimum | 1.4 ± 2.9 | 0.1 ±1.9 | 0.031 |
| **Aortic root surgery** |  |  |  |
| CuCo minimum | -1.9 ± 1.2 | -0.5 ± 2.4 | 0.001 |
| CuCo mid | 2.2 ± 1.4 | -0.1 ± 2.2 | <0.001 |
| CuCo max | -1.7 ± 1.4 | -0.1 ± 2.8 | 0.017 |
| CuCu mid | -2.0 ± 1.4 | -0.6 ± 2.2 | 0.004 |
| CuCu max | -1.6 ± 1.7 | -0.5 ± 1.7 | 0.015 |
| **Syndrome** |  |  |  |
| CuCo minimum | 0.5 ± 2.4 | 1.9 ± 1.2 | 0.036 |
| CuCo mid | 0.6 ± 2.3 | 2.0 ± 1.2 | 0.041 |
|  | **r^2^** | **Formula** | ***p*** |
| **Observer 1** |  |  |  |
| **Asymmetry index** |  |  |  |
| CuCo mid | 0.1629 | 26.23 ± 9.174 | 0.006 |
| CuCo max | 0.1323 | 25.36 ± 10.02 | 0.015 |
| CuCu min | 0.2006 | -10.3 ± 2.591 | <0.001 |
| CuCu mid | 0.1162 | 17.82 ± 7.83 | 0.024 |
| **Observer 2** |  |  |  |
| **Maximum diameter aortic root** |  |  |  |
| CuCu mid | 0.51 | -0.14 ± 0.04 | 0.002 |
|  |  |  |  |

Abbreviations: 3D self nav = 3D self-navigated high-resolution free-breathing whole heart, BAV = bicuspid aortic valve, CuCo = cusp to commissure, CuCu = commissure to commissure, max = maximum, min = minimal, SD = standard deviation

| **Table S6** Univariate analyses of parameters influencing intra-observer variability | | | |
| --- | --- | --- | --- |
|  | **r^2^** | **Formula** | ***p*** |
| **Observer 1** |  |  |  |
| **Maximum diameter aortic root** |  |  |  |
| 2D cine CuCu mid | 0.011 | 0.055 ± 0.02 | 0.024 |
| 3D self nav CuCo mid | 0.157 | -0.083 ± 0.029 | 0.007 |
|  | **No** | **Yes** | ***p*** |
| **Observer 1** |  |  |  |
| **Aortic root surgery** |  |  |  |
| 2D cine Cuco min | 0.6 ± 1.6 | 0.2 ± 1.4 | 0.033 |
| 3D self nav CuCu mid | 0.5 ± 1.7 | 0.8 ± 0.7 | 0.020 |
|  |  |  |  |
| **Observer 2** |  |  |  |
| **Syndrome** |  |  |  |
| 3D self nav CuCu minimum | -0.7 ± 0.6 | 0.1 ± 1.3 | 0.044 |
|  |  |  |  |

For abbreviations see table S5

| **Table S7** Univariate analyses of parameters influencing inter-observer variability |
| --- |

|  | **No** | **Yes** | ***p*** |
| --- | --- | --- | --- |
| **BAV** |  |  |  |
| 2D cine CuCu minimum | -3.2 ± 3.9 | -0.9 ± 1.6 | 0.003 |
| **Syndrome** |  |  |  |
| 2D cine CuCo maximum | 1.5 ± 1.5 | 0.5 ± 1.4 | 0.044 |
|  | **R^2^** | **Formula** | ***p*** |
| **Asymmetry index** |  |  |  |
| 3D self nav CuCu minimum | 0.497 | 19.85 ± 4.7 | 0.005 |
|  |  |  |  |

For abbreviations see table S5
